# Association among Complement Factor H Autoantibodies, Deletions of *CFHR*, and the Risk of Atypical Hemolytic Uremic Syndrome

**DOI:** 10.3390/ijerph13121209

**Published:** 2016-12-05

**Authors:** Hong Jiang, Meng-Nan Fan, Min Yang, Chao Lu, Ming Zhang, Xiao-Hong Liu, Le Ma

**Affiliations:** 1 The First Affiliated Hospital, Xi’an Jiaotong University College of Medicine, 277 Yanta West Road, Xi’an 710061, China; jiangh2015@stu.xjtu.edu.cn; 2 Key Laboratory of Shaanxi Province for Craniofacial Precision Medicine Research, College of Stomatology, Xi’an Jiaotong University, Xi’an 710004, China; 3 School of Public Health, Xi’an Jiaotong University Health Science Center, 76 Yanta West Road, Xi’an 710061, China; fanfan123@stu.xjtu.edu.cn (M.-N.F.); yangmin1993@stu.xjtu.edu.cn (M.Y.); 4 Xi’an Honghui Hospital, 555 Friendship Road, Xi’an 710054, China; luchao0925@163.com; 5 Key Laboratory of Environment and Genes Related to Diseases (Xi’an Jiaotong University), Ministry of Education of China, Xi’an 710061, China

**Keywords:** complement factor H-related genes, complement factor H, atypical hemolytic uremic syndrome, meta-analysis

## Abstract

To evaluate the association among complement factor H-related (*CFHRs*) gene deficiency, complement factor H (CFH) autoantibodies, and atypical hemolytic uremic syndrome (aHUS) susceptibility. EMBASE, PubMed, and the ISI Web of Science databases were searched for all eligible studies on the relationship among *CFHRs* deficiency, anti-FH autoantibodies, and aHUS risk. Eight case-control studies with 927 cases and 1182 controls were included in this study. *CFHR1* deficiency was significantly associated with an increased risk of aHUS (odds ratio (OR) = 3.61, 95% confidence interval (95% CI), 1.96, 6.63, *p* < 0.001), while no association was demonstrated in individuals with only *CFHR1/R3* deficiency (OR = 1.32, 95% CI, 0.50, 3.50, *p* = 0.56). Moreover, a more significant correlation was observed in people with both FH-anti autoantibodies and *CFHR1* deficiency (OR = 11.75, 95% CI, 4.53, 30.44, *p* < 0.001) in contrast to those with only *CFHR1* deficiency. In addition, the results were essentially consistent among subgroups stratified by study quality, ethnicity, and gene detection methods. The present meta-analysis indicated that *CFHR1* deletion was significantly associated with the risk of aHUS, particularly when combined with anti-FH autoantibodies, indicating that potential interactions among *CFHR1* deficiency and anti-FH autoantibodies might impact the risk of aHUS.

## 1. Introduction

Atypical, non-diarrhea-associated hemolytic uremic syndrome (aHUS) is an aggressive, fatal systemic disease, which is characterized by thrombocytopenia, microangiopathic hemolysis, and acute renal injury [1]. The main populations affected are children and young adults. In comparison with typical hemolytic uremic syndrome, aHUS has a higher risk of permanent renal failure and poorer prognosis [2]. It is reported that 25% of patients died in the acute phase, and up to 50% of patients who progress to end-stage renal disease (ESRD) need renal transplantation or dialysis for the remainder of their life [3,4]. Therefore, investigating the associated relevant risk factors may contribute to designing new diagnostic approaches and therapeutic guidelines.

The dysregulation of the alternative pathway of the complement cascade has been confirmed to play a pivotal role in the development of aHUS [5]. Mutations, polymorphisms, deficiency, or rearrangements in gene coding for various complement proteins have been reported in aHUS patients [6]. As the main complement protein, complement factor H (CFH) mediates the elimination of central complement activation component C3b [7]. The available data indicated that the mutations in CFH were linked to uncontrolled activation of the complement alternative pathway (CAP). Cell surface lack of control of the CAP could lead to CAP over-activation, which promotes microthrombi formation within small blood vessels, thereby resulting in tissue ischemia of the kidney [8,9]. Previous studies have revealed that CFH activity was partly modulated by factor H-related (FHR) proteins [10]. *CFHRs* (*CFHR1-5*) encoding FHR proteins and manifesting high degrees of sequence identity with CFH indicated that mutations in *CFHRs* may be partly responsible for the function abnormalities of CFH [11]. Several studies have investigated the association between *CFHR* deficiency and the pathogenesis of aHUS [12,13]. Although some studies have found an increased risk with *CFHR1* deficiency, others have failed to find this correlation [14,15]. Furthermore, it has been proposed that anti-FH autoantibodies together with *CFHR1* deficiency took part in the formation of CFH dysfunction, indicating that both autoantibodies and *CFHR1* absence might participate in the occurrence of aHUS. However, there still remains a need to attach importance to the effect of interactions among autoantibodies of the complement factors and CAP regulators on the development of aHUS [16,17]. 

Therefore, we conducted a meta-analysis of all eligible studies to estimate the association among *CFHRs* deficiency, anti-FH autoantibodies, and aHUS risk.

## 2. Materials and Methods

### 2.1. Search Strategy

A systematic literature search of PubMed, EMBASE, and the ISI Web of Science databases was performed up to March 2016. The following search terms and key words were used: (“*CFHRs*” or “complement factor H-related protein” or “FH-related proteins” or “anti-FH” or “factor H antibodies” or “anti-complement factor H autoantibody”) and (“aHUS ”or “atypical hemolytic uremic syndrome or atypical HUS”). There were no language restrictions. In addition, we manually searched the reference lists and pertinent reviews of retrieved articles for published or unpublished data. When datasets were incomplete for the required data, ongoing investigations or related experts were contacted for more information.

### 2.2. Study Selection

An initial screen of abstracts and titles was performed to identify all potential retrieved articles. Thereafter the full text was reviewed to determine whether the articles were suitable for this systematic review. Articles were eligible for inclusion in this study if they fulfilled the following criteria: (1) the study objective was to evaluate the relationship among *CFHRs* deficiency, anti-FH autoantibodies, and aHUS; (2) the original article was published as a cohort, case-control, or cross-sectional study; (3) the article provided the odds ratio (OR), relative risk (RR), or hazard ratio (HR) with a 95% confidence interval (95% CI) or sufficient data was provided to calculate it. If the articles reported on the same or overlapping data, only the article with the most updated data was included in this study. Three investigators (Hong Jiang, Meng-Nan Fan, and Min Yang) identified the eligible studies independently. Any differences in opinion were resolved through consensus with a third author (Le Ma) for adjudication.

### 2.3. Data Extraction and Quality Assessment

The following data were extracted using a standardized data extraction form that included: first author’s name, publication year, country of origin, study design, characteristics of the study population (number of aHUS cases and controls, origin of comparison group), gene and protein detection methods. If a study provided several risk estimates, only the most completely adjusted estimate was extracted. The quality of studies was evaluated based on the Newcastle-Ottawa Quality Assessment Scale (NOS) [18]. Each article was estimated with three domains (eight aspects): four criteria regarding the selection of participants (case definition, representativeness of the cases, selection of controls, and definition of controls), one item relating to comparability (comparability of cases and controls on the basis of the design or analysis), three items relating to outcomes (ascertainment of exposure, same method of ascertainment for cases and controls, and comparison of nonresponse rate between cases and controls). For each study, a maximum of one star can be given for each numbered criterion within the selection and outcome categories, and a maximum of two stars can be given for comparability. The quality scores of studies range from zero (worst) to nine (best). When a study’s score was ≥5, it was considered high quality, otherwise, it was considered low quality. Three authors (Hong Jiang, Meng-Nan Fan, and Min Yang) extracted data and assessed study quality independently. Discrepancies were resolved through discussion with a third author (Le Ma).

### 2.4. Statistical Analysis

The relative risk (RR) was considered as a measure to estimate the strength of the association across studies in principle. For case-control studies, relative odds ratios (OR) were considered as a surrogate estimate of the corresponding RR. Because the absolute risk of aHUS is low, the OR could approximate the RR. If articles did not report RR, we used the number of events in each group to calculate the RR for each study and used them for pooling analyses. In this meta-analysis, pooled RR and 95% CI were used to estimate the strength of the association among *CFHRs*, anti-FH autoantibodies, and aHUS, using a fixed effects model or a random effects model according to the heterogeneity. Heterogeneity between studies was examined by calculating Cochran’s Q statistic and the I^2^ test (*p* value less than 0.10 was considered to indicate heterogeneity and I^2^ > 50% indicated a statistical significance). When significant statistical heterogeneity was detected, a stratified analysis was conducted to explore the potential source of heterogeneity. The variables included in the stratified analyses were study quality, ethnicity, and gene detection methods. In addition, sensitivity analyses were performed to evaluate the effect of each individual study by excluding each study from the meta-analysis. Egger’s weighted regression method, Begg’s rank correlation method, and visual inspection of funnel plots were used to assess the publication bias [19,20]. All analyses were performed using Stata version 11.0 (Stata Corp LP, College Station, TX, USA).

## 3. Results

**Figure 1:**
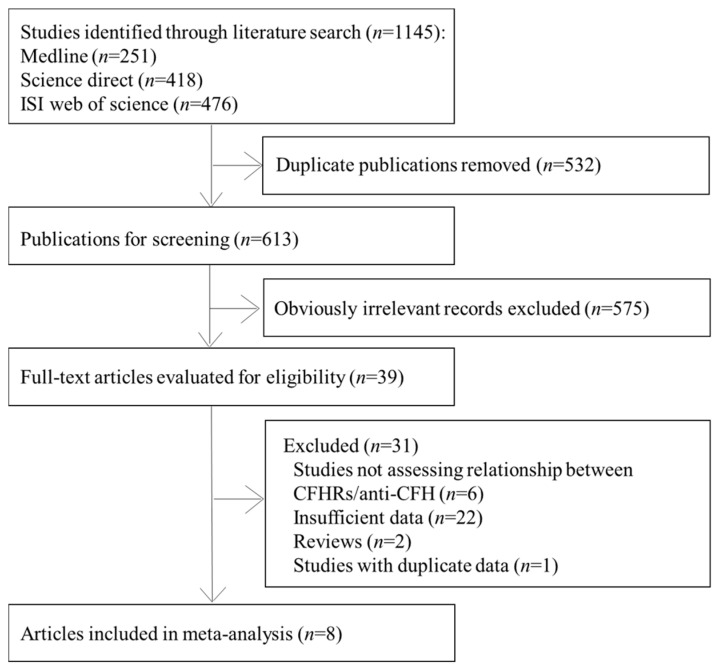
Flowchart for the selection of eligible studies.

A total of 1145 articles were retrieved through comprehensive searching. After excluding duplicates and irrelevant references, 39 records were left for full text and data effectiveness review, and eight articles (nine studies) were included in this meta-analysis [10,13,14,15,17,21,22,23] (Figure 1).

### 3.1. Characteristics of the Studies

The characteristics of the included studies are presented in Table 1. All eligible studies were case-control designed, comprising a total of 1065 cases and 1266 controls. Seven studies were carried out in Western countries and two in Asian countries. Six of the included studies used population-based (PB) controls, one investigation used hospital-based (HB) controls, and the remaining one used PB + HB controls. Six of nine studies detected *CFHR* deficiency through multiplex ligation-dependent probe amplification. The other three studies used polymerase chain reaction, comparative genomic hybridization, or Western blot, respectively. Six of the eight studies detected anti-FH autoantibodies in serum samples using enzyme-linked immunosorbent assay. Furthermore, quality assessment revealed that six studies scored ≥5 and were classified as high quality.

### 3.2. CFHR1 Deficiency and aHUS Risk

**Figure 2:**
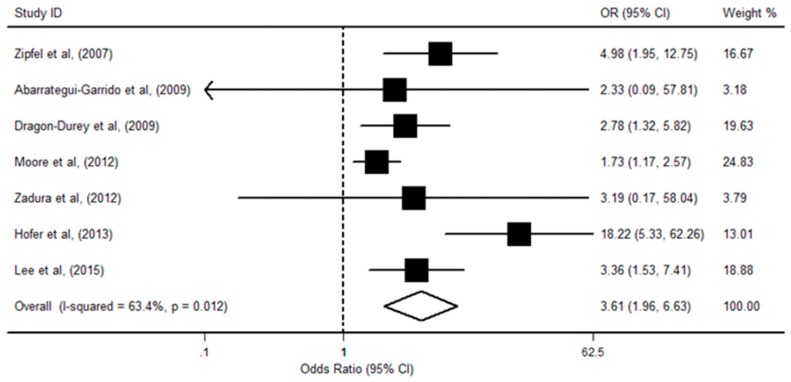
Forest plot on the association between *CFHR1* deficiency and aHUS risk. For each study, the estimation of OR and its 95% confidence interval (95% CI) are plotted with a box and a horizontal line. The pooled odds ratio is represented by a diamond. The area of the black squares reflects the weight of the study in the meta-analysis.

The relationship between *CFHR1* deficiency and the risk of aHUS was evaluated in seven studies, including 832 cases and 1028 controls. Among the studies, five reported a statistically significant relationship. There was significant heterogeneity (I^2^ = 63.4%; *p* = 0.01) between the data sets, and the random-effects model was used to calculate the pooled OR. The pooled results showed that *CFHR1* deficient individuals had a higher susceptibility to aHUS by approximately 2.6-fold in comparison with no-deficiency patients (OR = 3.61; 95% CI, 1.96, 6.63; *p* < 0.001; Figure 2). The result of stratified analyses by study quality and ethnicity showed that these factors did not alter the association direction and magnitude of the overall effect. The meta-regression analysis showed that gene detection methods were associated with an increased risk of aHUS (Table 2). The sensitivity analysis showed that the significant association among the pooled OR remained stable between *CFHR1* deficiency and aHUS. In addition, there was no obvious asymmetry of funnel plots, and no evidence of publication bias was detected among studies (Begg’s test *p* = 0.76; Egger’s test *p* = 0.16).

### 3.3. CFHR1/R3 Deficiency with aHUS Risk

**Figure 3:**
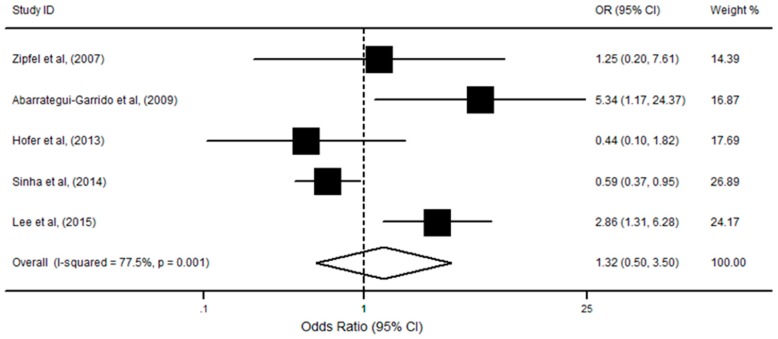
Forest plot on the association between *CFHR1/R3* deficiency and aHUS risk. For each study, the estimation of OR and its 95% CI are plotted with a box and a horizontal line. The pooled odds ratio is represented by a diamond. The area of the black squares reflects the weight of the study in the meta-analysis.

Five studies reported data on the association between only *CFHR1/R3* deficiency (without anti-FH autoantibodies) and aHUS risk, with a total of 477 cases and 436 controls. Three reported a slight association of such deficiency with aHUS incidence. Findings from the current analysis showed that there was no significant association among *CFHR1/R3* absence and the risk of aHUS (OR = 1.32; 95% CI, 0.50, 3.50; *p* = 0.56; Figure 3), using the random-effects model (I^2^ = 77.5%; *p* = 0.001). Subsequently, we also performed stratified analysis by study quality, ethnicity, and gene detection methods. The result showed that these factors did not alter the association direction and magnitude of the overall effect. Moreover, no publication bias was detected and the funnel plots were symmetric upon visual inspection (*p* = 0.46), which is confirmed by the Egger’s test (*p* = 0.35).

### 3.4. CFHR1 Deficiency, Anti-CFH, and aHUS Risk

**Figure 4:**
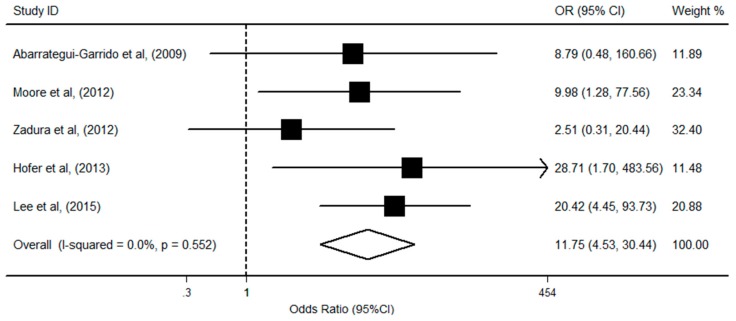
Forest plot on the association among *CFHR1* deficiency, anti-FH autoantibodies, and aHUS risk. For each study, the estimation of OR and its 95% CI are plotted with a box and a horizontal line. The pooled odds ratio is represented by a diamond. The area of the black squares reflects the weight of the study in the meta-analysis.

We subsequently evaluated the effect of anti-FH autoantibodies with *CFHR1* deficiency on the risk of aHUS in five studies, which included 545 cases and 377 controls. Of the included studies, three showed that both *CFHR1* deficiency and autoantibody had a positive relationship with aHUS risk. There was no evidence of statistical heterogeneity among the eligible studies (I^2^ = 0.0%, *p* = 0.55), and the fixed-effects model showed that individuals with *CFHR1* deletion and anti-FH autoantibodies had an obviously increased risk of aHUS (OR = 11.75; 95% CI, 4.53, 30.44; *p* < 0.001; Figure 4). In addition, neither the Egger’s test (*p* = 0.77) nor the Begg’s test (*p* = 0.46) revealed significant publication bias.

## 4. Discussion

The results from this comprehensive meta-analysis demonstrated that the deletion of *CFHR1* was significantly associated with aHUS. Additionally, further analysis found a stronger association in patients with both *CFHR1* deletion and anti-FH autoantibodies in comparison with only *CFHR1* deficiency, indicating that *CFHR1* deficiency might integrate with anti-FH autoantibodies, which contributes to the development of aHUS.

Insufficient inhibition of complement is associated with a quantity of autoimmune and inflammatory diseases’ pathological processes such as aHUS [23,24]. Several studies have suggested that CFH plays a key role in maintaining complement homeostasis through inhibiting the formation and accelerating the decay of C3-convertase or acting as a cofactor to complement factor I (CFI) in the degradation of C3b in the alternative pathway [25,26]. Moreover, as the major components of a complement system, CFH activities were modulated by other factors consisting of FHRs. Both CFH and FHRs (FHR1-5) belong to human chromosome 1q32 and reside in the regulators of complement activation (RCA) gene cluster [11,27]. *CFHRs* do encode proteins (FHR1-5) that are likely to modify the function of CFH and regulate complement activity, which would maintain the balance of the complement system and prevent humans from damaging aHUS. In concordance with previous studies that reported *CFHR1* deletion was relevant to aHUS susceptibility, the results of present meta-analysis showed a significant association between *CFHR1* deficiency and an increased risk of aHUS. The potential mechanism of FHR1 on complement activation and regulation might be via its ability to mediate CFH activity [28]. Under normal circumstances, alternative pathway discrimination between activator and non-activator surfaces is mediated by CFH, CFH can use the heparin/gycosaminoglycan site on short consensus repeats (SCRs) 20 for binding to endothelial cells and to C3b and its cleavage fragment C3d [29,30,31], which has high homology with FHR4-5. Nevertheless, the auto-antigenic epitope of CFH and its homologous site in FHR1 are structurally different [30]. Therefore, *CFHR1* deficiency may induce auto-antigenic neoepitope which is related to the formation of autoantibodies, and reduces the function of CFH. Meanwhile, subtotal CFH insufficiency can give rise to aHUS after a complement-activating trigger within the kidney and that the latter is C5 dependent [32,33]. Moreover, FHRs could influence complement activation and regulation through efficiently competing with CFH for binding to the specific surface. The abnormal multimeric structure of FHR1 proteins likely contributes to the cell surface dysregulation [29]. Cell surface lacking control of the CAP would lead to its over-activation and promote microthrombi formation within small blood vessels. Such microthrombi will injure the red blood cells and cause kidney tissue ischemia [8,33]. Moreover, *CFHRs* and CFH have several independent large genomic duplications, such repeats tend to occur in nonallelic homologous recombinations, which results in the loss of *CFHR1* [34,35]. FHR1 deficiency has a potential impact on CFH binding to the CAP convertase or the regulatory function of the terminal pathway, which is related to the occurrence of aHUS [8,35]. In addition, a regulatory function of FHR1 on the C5 convertase or terminal pathway has recently been reported, which might also contribute to aHUS pathogenesis [36]. In contrast with the significant association between *CFHR1* deficiency and the increased risk of aHUS, the present studies showed that individuals carrying *CFHR1/R3* deficiency (without autoantibodies) had no significant association with aHUS. It is possibly influenced by the small number of included studies. Thus, further multi-centric studies investigating the relationship between aHUS and different combinations of *CFHRs* deficiency need to be conducted before definitive conclusions can be made.

Evidence from a previous study found that autoantibodies to CFH were connected with the development of aHUS [37]. The epitope of the autoantibody is located on the C-terminal SCRs 19–20 of CFH, which is responsible for recognition and binding to C3b and to cellular surfaces. Blocking the SCRs 19–20, anti-FH autoantibodies could impair the binding of factor H to C3b and be associated with uncontrolled complement activation at the cell surface and with hemolysis [31]. Results from the present study indicated that both anti-FH autoantibodies and *CFHR1* deficiency were significantly associated with the increased risk of aHUS, which suggests that this autoantibody might cross-react with *CFHR1* deficiency to promote the progress of aHUS. The underlying mechanism for such an observed relationship might be that such a deficiency may favor development of these specific autoantibodies. CFH and FHR1 have high degrees of sequence duplications, especially in SCRs 19–20 and SCRs 4–5 (autoantibody epitope site) [35,38]. Such a special gene structure would cause a failure of central and/or peripheral tolerance and induce the anti-FH autoantibodies, resulting in increased progression of kidney injury [39,40].

Findings from this meta-analysis have important public health significance. Cases of aHUS generally have a poor outcome [2,41]. Therefore, early specific diagnosis and treatment might lead to favorable outcomes. The central pathophysiology of aHUS is uncontrolled activation of the complement pathway. The finding of a significant relationship between aHUS and *CFHRs* deficiency and/or autoantibodies potentially has important clinical implications for investigating these risk factors in the early stage of aHUS and improving prognosis. Though the result from Holmes et al. suggests that *CFHR1/R3* deletion in the normal population is different worldwide. Depending on ethnic origin, the highest frequency for the deletion (54.7%) was seen in Nigeria [42]. *CFHRs* deficiency and autoantibodies could also be considered as a surrogate biomarker for some clinically relevant incidents or aHUS susceptibility, especially in designing new diagnostic approaches, identifying susceptible people, and monitoring the treatment effects.

Several potential limitations should be also considered in interpreting the results of this meta-analysis. First, as no available cohort studies were found, this meta-analysis only extracted data from case-control studies. Retrospective study is subjected to internal methodological deficiencies, which may limit the power of this meta-analysis. Second, our results were based on unadjusted estimates, potential confounding components such as pregnancy, family history, drugs, other complement factor genes, and other genetic abnormalities [43] likely affect the risk of aHUS, and studies in this meta-analysis did not control for these factors or provide sufficient data to analyze the association among *CFHRs* deficiency, anti-FH autoantibodies, and aHUS adjusted for these covariates. Thereby, other complement factors, genetic abnormalities, or other confounding factors might affect the results of the present analysis. Nevertheless, the qualities of most of the eligible studies were high, which adds to the strength of our analysis. Third, the present results were also likely to be affected by different separate examination methods of *CFHR1*. The way to examine the *CFHR1* status of the patients and controls, varies from one study to another, and that might affect the present results as a potential confounding factor. Furthermore, one study used western blot to identify *CFHR1* deficiency. As aHUS could be treated by administration of blood derived products, the approach by western blotting might be at high risk of false results in aHUS. Although this study should not substantially affect the overall qualitative inference, we cannot exclude the potential influence which might have led to underestimation or overestimation of the association. Fourth, the pooled results were mainly conducted in Western countries, which limited the generalization of findings. Last, although statistical tests did not suggest the presence of publication bias for the present study, we could not exclude all publication bias.

## 5. Conclusions

The present meta-analysis provides evidence of the association between *CFHR1* deletion, anti-FH autoantibodies and aHUS. *CFHR1* deficiency was significantly associated with increased risk of aHUS. Moreover, this study also identified potential interactions between anti-FH autoantibodies and *CFHR1* deletion in aHUS patients, indicating that the development of aHUS could be exacerbated via interaction between *CFHR1* deficiency and anti-FH autoantibodies. 

## Figures and Tables

**Table 1:** Characteristics of studies included in this meta-analysis.

First Author (*y*)	Country of Origin	Study Type	Sample Size (Cases)	Sample Size (Controls)	Source of Controls	Gene Detection Methods	Protein Detection Methods	Study Quality *
Lee et al., 2015	Korea	Case-Control	51	100	HB	MLPA	ELISA	High
Sinha et al., 2014	Indian	Case-Control	138	84	PB	MLPA	Western blot	High
Hofer et al., 2013	European	Case-Control	116	118	PB	CGH	ELISA	Low
Moore et al., 2012	UK	Case-Control	142	100	PB	MLPA	ELISA	High
Zadura et al., 2012	German	Case-Control	103	345	PB	-	ELISA	High
Abarrategui-Garrid et al., 2009	Spain	Case-Control	151	230	HB + PB	MLPA	ELISA	High
Dragon-Durey et al., 2009	France	Case-Control	177	70	PB	MLPA	ELISA	High
Zipfel et al., 2007	Germany	Case-Control	121	100	PB	PCR	NR	Low
Zipfel et al., 2007	UK	Case-Control	66	119	PB	MLPA	Western Blot	Low

aHUS, Atypical hemolytic uremic syndrome; HB, Hospital-based; PB, Population-based; MLPA, Multiplex ligation-dependent probe amplification; CGH, Comparative genomic hybridization; ELISA, Enzyme linked immunosorbent assay; PCR, polymerase chain reaction; NR, Not reported; * Study quality was judged based on Newcastle-Ottawa Scale.

**Table 2:** Meta-analysis of the association between the *CFHR1* deficiency/*CFHR1/R3* deficiency and atypical hemolytic uremic syndrome risk.

Subgroup	*N*	Pooled OR	95% CI	*p*	
				Heterogeneity	Meta-Regression
*CFHR1* Deficiency	-	-	-	-	-
Study Quality	7	-	-	-	0.059
High	5	2.12	1.55, 2.90	0.572	
Low	2	3.61	1.96, 6.63	0.095	
Ethnicity	-	-	-	-	0.703
Caucasians	6	1.88	1.22, 2.54	0.005	
Asians	1	3.36	0.42, 6.30	NA	
Gene Detection Methods	6	-	-	-	-
MLPA	5	2.58	1.70, 3.91	<0.001	0.001
CGH	1	18.22	5.33, 62.26	NA	
*CFHR1/R3* Deficiency	-	-	-	-	-
Study Quality	5	-	-	-	0.763
High	4	1.35	0.44, 4.15	0.001	
Low	1	1.25	0.20, 7.61	NA	
Ethnicity	5	-	-	-	0.694
Caucasians	3	1.41	0.31, 6.52	0.06	
Asians	2	1.26	0.27, 5.09	0.001	
Gene Detection Methods	5	-	-	-	-
MLPA	3	1.86	0.47, 7.35	0.376	0.461
CGH	1	0.44	0.10, 1.82	NA	
PCR	1	1.25	0.20, 761	NA	

OR, odds ratio; 95% CI, 95% confidence interval; MLPA, Multiplex ligation-dependent probe amplification; CGH, Comparative genomic hybridization; PCR, polymerase chain reaction. NA, Not Available.
